# Vaccination Coverage and Compliance with Three Recommended Schedules of 10-Valent Pneumococcal Conjugate Vaccine during the First Year of Its Introduction in Brazil: A Cross-Sectional Study

**DOI:** 10.1371/journal.pone.0128656

**Published:** 2015-06-10

**Authors:** Fabricia Oliveira Saraiva, Ruth Minamisava, Maria Aparecida da Silva Vieira, Ana Luiza Bierrenbach, Ana Lucia Andrade

**Affiliations:** 1 Department of Community Health, Institute of Tropical Pathology and Public Health, Federal University of Goias, Goiania, Brazil; 2 Department of Pediatric, School of Nursing, Federal University of Goias, Goiania, Brazil; 3 Nursing, Physical Therapy and Nutrition Department, Pontifical Catholic University, Goiania, Brazil; 4 Research and Education Institute (IEP), Hospital Sírio-Libanês, São Paulo, Brazil; London School of Hygiene and Tropical Medicine, UNITED KINGDOM

## Abstract

Pneumococcal 10-valent conjugate vaccine (PCV10) was introduced to Brazil’s National Immunization Program (NIP) in 2010. During the first year of vaccine introduction three schedules were used to deal with age at initiation of PCV for catch-up purposes: 3 primary doses + 1 booster (for children aged ≤6 months), a catch-up schedule of 2 doses + 1 booster (7-11 months), and a catch-up schedule of a single dose (12-15 months). The purpose of this study was to assess the magnitude and associated risk factors for under-vaccination or lack of on time vaccination six to eight months after PCV10 introduction. A household survey was conducted in the municipality of Goiania with 1,237 children, who were retroactively classified into one of three age groups, as a factor of the child’s age relatively to 30 days after PCV10 introduction. Socioeconomic characteristics and vaccination dates were obtained during home interviews. Vaccination coverage was defined as the percentage of children who completed the recommended number of doses. Compliance with recommended schedules was defined as the percentage of children who received all valid doses at the NIP recommended time interval. Adjusted prevalence ratios (PR) of variables independently associated with coverage and compliance were estimated by log binomial regression. Coverage of DTP-Hib was used for comparison purposes. Overall, vaccination coverage was 54.6% (95% CI 52.1-57.7%), lower than DTP-Hib coverage (93.0%; 95% CI 91.5-94.3%). Compliance with recommended schedules was 16.8% (95% CI: 14.7-18.6%). Children 7-11 months old had lower coverage (40.7%) and compliance (6.3%) compared to children aged 12-15 months (coverage: 88.8%; compliance: 35.6%) and ≤6 months old (coverage: 54%; compliance: 18.8%). Having private health insurance was associated with higher PCV10 coverage (PR=1.25; 95% CI: 1.06-1.47, p=0.007), and compliance (PR=1.09; 95% CI: 1.02-1.16, p=0.015). Although PCV10 coverage rapidly increased shortly after vaccination introduction, it was not matched by compliance with recommended schedules. Public initiatives should target compliance of PCV10 because of the burden of pneumococcal diseases on childhood morbidity and mortality.

## Introduction

Infections caused by *Streptococcus pneumoniae* are a major cause of morbidity and mortality worldwide, being the leading cause of bacterial pneumonia, meningitis, and sepsis in children [1]. In developing countries, children living in lower socioeconomic conditions are at higher risk for pneumococcal diseases, especially pneumonia [2–4]. To reduce the burden of such diseases, the inclusion of pneumococcal conjugate vaccines in childhood immunization programs has been recommended as a priority strategy by the World Health Organization/WHO [5]. Following the WHO recommendation, Brazil’s National Immunization Program (NIP) included 10-valent pneumococcal conjugate vaccine (PCV10) in the routine immunization calendar free of charge for all children on June 2010. This vaccine includes 1, 4, 5, 6B 7F, 9V, 14, 18C, 19F, and 23F [5]. The 13 valent pneumococcal vaccine (PCV13) is also available in Brazil at private services only, and includes all serotypes in PCV10, with the addition of 3, 6A and 19A [5].

Providing vaccines free of charge has been demonstrated as a successful measure for Brazil’s NIP, as children vaccinated exclusively from public immunization providers have higher vaccination coverage [6]. Yet, introducing PCV10 to Brazil’s routine immunization adds a new shot to a childhood schedule that already includes a considerable number of injections [7], which could jeopardize parents willingness to vaccinate [8, 9].

The NIP coverage rates are usually estimated using the number of administered doses as registered in administrative data, which also allows for estimating coverage homogeneity (number of municipalities that have reached 95% vaccination coverage divided by the total number of municipalities multiplied by one hundred) [10, 11]. These estimates evaluate one aspect of vaccination recommendations, that is, completing the number of doses. However, there are other aspects included in vaccination recommendations as a whole, which are usually left out, such as the minimum and recommended age to start vaccination, the age groups for which the vaccine should be routinely administered, the minimum and recommended interval between scheduled doses, vaccine contraindications, among others [12]. Vaccination recommendations for doses, ages and time intervals are also frequently updated as evidence is generated [13] to ensure that vaccination provides maximum effectiveness against vaccine-preventable diseases [14]. Thus, assessing compliance with other aspects related to vaccination recommendations (and not only the administered number of doses) is highly desirable.

We conducted a survey to investigate both coverage and compliance six to eight months after the introduction of PCV10 into the vaccination routine in Goiania, a developed municipality of Brazil [15]. We also aimed to identify potential factors associated with coverage and compliance. The ultimate goal was to support Brazil’s NIP strategic planning and priorities, through the identification of vaccination gaps.

## Methods

### Study design and setting

This population-based household survey is part of a major ongoing research that aims to evaluate the effectiveness of PCV10 on pneumococcal carriage in Brazilian children. The survey was carried out in the municipality of Goiania (≈1,300,000 inhabitants), Midwestern Brazil from December 2010 to February 2011. In 2010, 33,780 inhabitants were younger than 2 years old; infant mortality was estimated to be 12.6/1,000 live births; and the Municipal Human Development Index was 0.799 [15].

Sixty-five public immunization rooms and twenty-six mobile teams are distributed over the seven health districts of the municipality of Goiania among family-health services, day-clinics, hospitals and emergency units. There are also two Reference Centers for Special Immunization. All immunization rooms are open from Monday to Friday, 8:00am to 6:00pm. Administration of vaccines is recorded both in the child’s vaccination card and also in Goiania’s immunization register immediately after vaccine administration, resulting in a real-time online database of individual-level vaccine uptake registered by all public immunization providers, which can be easily searched by vaccination staff, should the child’s parent or legal guardian come to the immunization room without the vaccination card. The staff also customarily writes down with a pencil the date of the next dose on the vaccine card, as a reminder of the due date. Prescription is not required for routine immunization. Both pediatricians and nurses may prescribe vaccines not included in routine immunization for children with special needs, which are administered only in Reference Centers. All vaccines are free of charge from public providers.

### PCV10 introduction and schedules

The introduction of PCV10 to routine immunization at municipality of Goiania started on June 14th, 2010. Three different schedules were put in place during PCV10 introduction period: 3 primary doses (plus a booster dose at 12–15 months) for children aged 6 months or younger, two primary doses (plus a booster dose at 12–15 month) for children between 7–11 months (catch-up schedule), and a single dose (no booster) for children 12–15 months (catch-up schedule). PCV10 was made available in public providers only.

Therefore, during the introduction of PCV10 in Brazil, a child had the opportunity to receive the first dose at any moment between 2 and 15 months of age, independently of whether the child had other due vaccines or not. Two national immunization days took place in Brazil after PCV10 introduction: one in June 20, and the other on September 19. Although the focus of these days was to vaccinate children against polio, it was also used to evaluate children’s card for due and past-due vaccination dates, and to administer any recommended/missed vaccine—including PCV10. Besides, visits to the health care service for other routine immunizations should also be used to start PCV10 vaccination. To this end, the Brazilian routine childhood schedule recommended at least six vaccination appointments in 2010. One at 2 and 4 months of age to receive DTP-Hib, polio, and the oral vaccine against human rotavirus. Another at 6 months of age to receive HepB, DTP-Hib, and polio. Other visits to the health care at 9 months of age (to receive the vaccine against yellow fever), at the first birthday (to receive MMRV), and at 15 months of age (to receive DTP and polio) [7]. In 2010, routine medical appointments for growth follow-up were recommended at the same ages of the immunization childhood schedule. Meningococcal C conjugate vaccine at 3, 5 and 15 months of age was also included in the routine childhood schedule on October 2010.

### Study population

**Fig 1:**
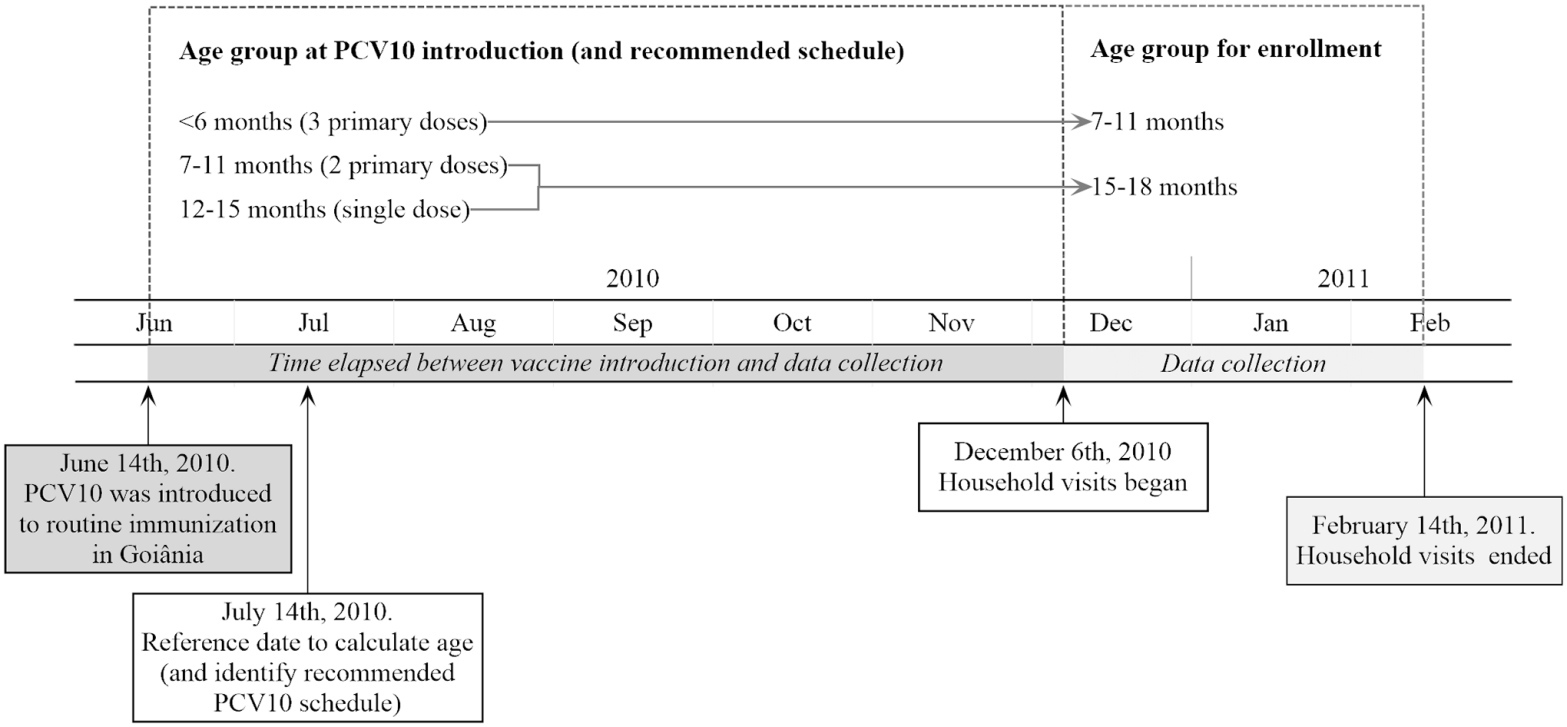
From PCV10 introduction to household visits: a timeline of study events.

The target population of the present study was children aged 7-11mo (n = 647) and 15-18mo (n = 590). This choice took into account the time interval of six to eight months elapsed between the introduction of the vaccine (June 2010) and the onset of data collection (December 2010-February 2011). The rationale was to include all three age groups (≤6 months, 7-11months, 12-15months) for which Brazil’s National Immunization Program recommended PCV10 (Fig 1).

### Sampling and sample size

A list of 25,656 study age group children within the catchment area (with addresses, names of mothers, gender, and dates of births) was obtained from Brazil’s Live Births Registry System. The list was sorted by gender, district of residence, and date of birth. A systematic sampling was applied to that ordered list; a sampling interval was calculated (*k* = 19) and a random starting point (13) was selected. The list was treated circularly, with a return to the top when the end of the list was reached. Sampling was proportional to age and district of residence in Goiania.

A sample size of 1,300 children was originally calculated for the purpose of the major investigation, to assess PCV10 effectiveness on pneumococcal carriage, which has been described elsewhere [16]. In order to evaluate if that sample size would suffice for the present study, we recalculated the sample size, considering the PCV coverage of 7.2% estimated by a previous survey [17], 2.0 design effect and ±3% error. We found that a sample size of 594 children would be necessary to assess vaccination coverage, which is less than half the number of 1,237 children included in the final sample of this study.

### Data collection

Household visits took place from December 2010 to February 2011, that is, between six and nine months after the introduction of PCV10 to local routine immunization (Fig 1). The date of each pneumococcal vaccine uptake was retrieved from the immunization card. A questionnaire was applied to obtain socioeconomic characteristics—type of health insurance, family’s annual income, mother’s education level, number of household members, number of children in the household, number of people sharing a bedroom with the child, and current or previous daycare attendance. If the immunization card were incomplete or unavailable, the child’s vaccination dates were retrieved from Goiania’s immunization register whenever possible. Children whose vaccination dates could not be retrieved were not included in the final sample, as well as those who died, those whose legal guardian could not be found, and those whose address as obtained on the Live Births Registry was no longer valid. We also collected the dates of DTP-Hib vaccine uptake from the child’s immunization card, as all three doses are recommend at the same ages of PCV10 primary schedule (2, 4 and 6 months old).

In order to retroactively classify children into one of the three mentioned age groups, we calculated each child’s age as of July 14th, 2010, that is, 30 days after PCV10 was introduced to local routine immunization program. Before PCV10 introduction, other pneumococcal vaccines were available either at public immunization providers (PCV7; free of charge for children with special vaccination requirements) or at private immunization services (PCV13; medical prescription required, usually for children from high socioeconomic stratum). Children who received a pneumococcal vaccine other than 10-valent fell under vaccination exceptions and therefore were not included in the final sample.

For children in the age group of ≤6 months old, we also estimated the date the child would ideally complete the three primary doses, should the child have received them according to recommended ages and within grace periods. We added to the date of birth 2months + 30 days (for the first dose), then 2 months + 7 days (for the second dose), then 2 months + 7 days (for the third dose). Any child that was recruited before the third dose ideal due date was not included in the final sample.

### Ethics Statement

Written informed consent was obtained from each participant’s parent(s) or legal guardian(s). The Ethics Committee of the Federal University of Goias approved the study (protocol # 145/2010).

### Outcome measures

Two outcomes were considered: vaccination coverage, and compliance with the recommended schedules. Vaccination coverage was defined as the percentage of children who completed the number of doses as recommended by the NIP (according to children’s age group) prior to household visits. Coverage was estimated to all children and to each age group / schedule and was presented in percentages. We also observed each child’s status for the number of doses: fully vaccinated (children who received all recommended doses, according to each age group’s recommendation), under-vaccinated (children who started the vaccination, but did not receive all recommended doses), and not vaccinated (children who did not start vaccination).

Because the NIP adopted three different schedules at the introduction of PCV10, compliance was evaluated separately for each schedule and for the first, second and third dose independently. For children aged ≤6 months (by July 14th, 2010, 30 days after vaccine introduction), the following definitions were used:

1st dose: a child was considered compliant if he/she received the dose by July 14th, 2010 (30 days after vaccine introduction) or up to two months of age + 30 days (if the child reached that age after July 14th, 2010).
2nd dose: a child was considered compliant if he/she received the dose between 24 days and 2 months (plus a grace period of 7 days) after the date of the 1st dose, regardless of whether the 1st dose was correct, delayed or too early.
3rd dose: a child was considered compliant if he/she received the dose from 24 days to 2 months (plus a grace period of 7 days) after the date of 2nd dose, regardless of whether the 2nd dose was correct, delayed or too early.

For children aged 7–11 months (by July 14th, 2010), the following definitions were used:

1st dose: a child was considered compliant if he/she received the dose up to 30 days after PCV10 introduction, regardless of the child’s age.
2nd dose: a child was considered compliant if he/she was vaccinated between 24 days to 2 months (plus a grace period of 7 days) after the date of the 1st dose, regardless of whether the 1st dose was correct, delayed or too early.

For children aged 12–15 months (by July 14th, 2010):

single dose: a child was considered compliant if he/she received the dose up to 30 days after PCV10 introduction, regardless of the child’s age. It is worth noting that, for this age group, the NIP did not recommend a second or a booster dose, and therefore, having received more than one dose was not considered for the analysis.

For all age groups the analysis took into account a grace period of 30 days after the introduction of PCV10 to start vaccination. It also included the recommended time interval between doses [18–20]. Six weeks was considered the minimum acceptable age for the first dose and four weeks was considered the minimum interval between doses [18]. Doses administered five days or earlier than the minimum age or time interval were considered invalid [13]. For each dose, being non-compliant indicated that the child was either receiving the scheduled dose later than expected, or that the child was receiving an invalid dose, or that the child was not vaccinated at all. Being compliant with the schedule meant that the child was compliant with all doses recommended by the NIP according to age group.

Overall compliance was estimated as the number of all compliant children divided by the total number of children in the sample, multiplied by 100, that is, the percentage of children who received all valid doses within the time interval recommended by the NIP [19, 20].

Although we did collect data of all doses the child had received (and not only the recommended ones), there was not enough time for all children to receive the booster dose, which was not included in the analysis. Some children in the age groups of 7–11 and 12–15 months old received more than the number of recommended primary doses (2 doses and a single dose, respectively), although none received more than 4 doses. These extra doses were computed, but not considered for the analysis; these children were considered to be fully vaccinated.

For comparison purposes, we estimated DTP-Hib coverage as the percentage of children who received the third dose of this vaccine. In order to look at missed opportunities, we identified children who started and finished DTP-Hib primary schedule from June 14th, 2010 on. Among those, we calculated how many completed PCV10 schedule, considering that the same visit to the health care service should be used to administer both vaccines. We also assessed whether PCV10 unvaccinated children had been to a health care service to receive DTP-Hib vaccine after PCV10 introduction.

### Data analysis

Data input and analysis were performed using Statistical Package for the Social Sciences (v.20.0). Descriptive statistics for receipt of first PCV10 dose were given: median age according to each age group, number of children who received the first dose before 12 months of age. The Kaplan-Meier estimator (1-survival) [21] was used to predict time to receive the first PCV10 dose. We considered the date of PCV10 introduction in the local immunization program (June 14th, 2010) as the starting point; receiving the first dose of PCV10 as the event; and the age at vaccine introduction (≤6 months, 7–11 months, 12–15 months) as the between-subjects factor. We censored unvaccinated children and those who had started PCV10 vaccination before June 14th, 2010. Survival time was counted as the number of days from PCV10 introduction to receipt of first dose. Breslow’s chi-square statistics was used to compare cumulative incidence of first PCV10 dose between age groups; p values <0.05 were considered significant. Medians of survival time for each age group were also estimated, with respective Standard error (SE) and 95% confidence interval.

A formal detection-tolerance or the variance inflation factor (VIF = 1/Tolerance) was used to detect collinearity. VIF was calculated by including all possible pairs of socioeconomic variables and each outcome individually in a linear regression model. Tolerance values below 0.4 suggested collinearity [22].

At univariate analysis, prevalence ratios and respective 95% confidence intervals (95%CI) for predictors were obtained using Pearson’s chi-square test. Explanatory variables significant to the 0.1 level at univariate analysis were added into a multivariable model to adjust for confounding factors. Log binomial models were used for the multiple regression analyses, in which we aimed at identifying multiple independent predictors for each of our outcome variables: (1) binary variable expressing vaccine coverage (“fully vaccinated”/“under or not vaccinated”) and (2) binary variable expressing compliance with recommended schedules (“compliant”/“delayed or no vaccinated”). The choice of log binomial models instead of logistic models was motivated by the expected high prevalence of the studied outcomes (coverage and compliance), and therefore the preference to express the measure of effect in terms of the more intuitively interpreted relative risk and not the odds ratio. Separate models were developed for each of the outcome variables. A *p-*value <0.05 was considered statistically significant. All tests were two-tailed.

## Results

**Fig 2:**
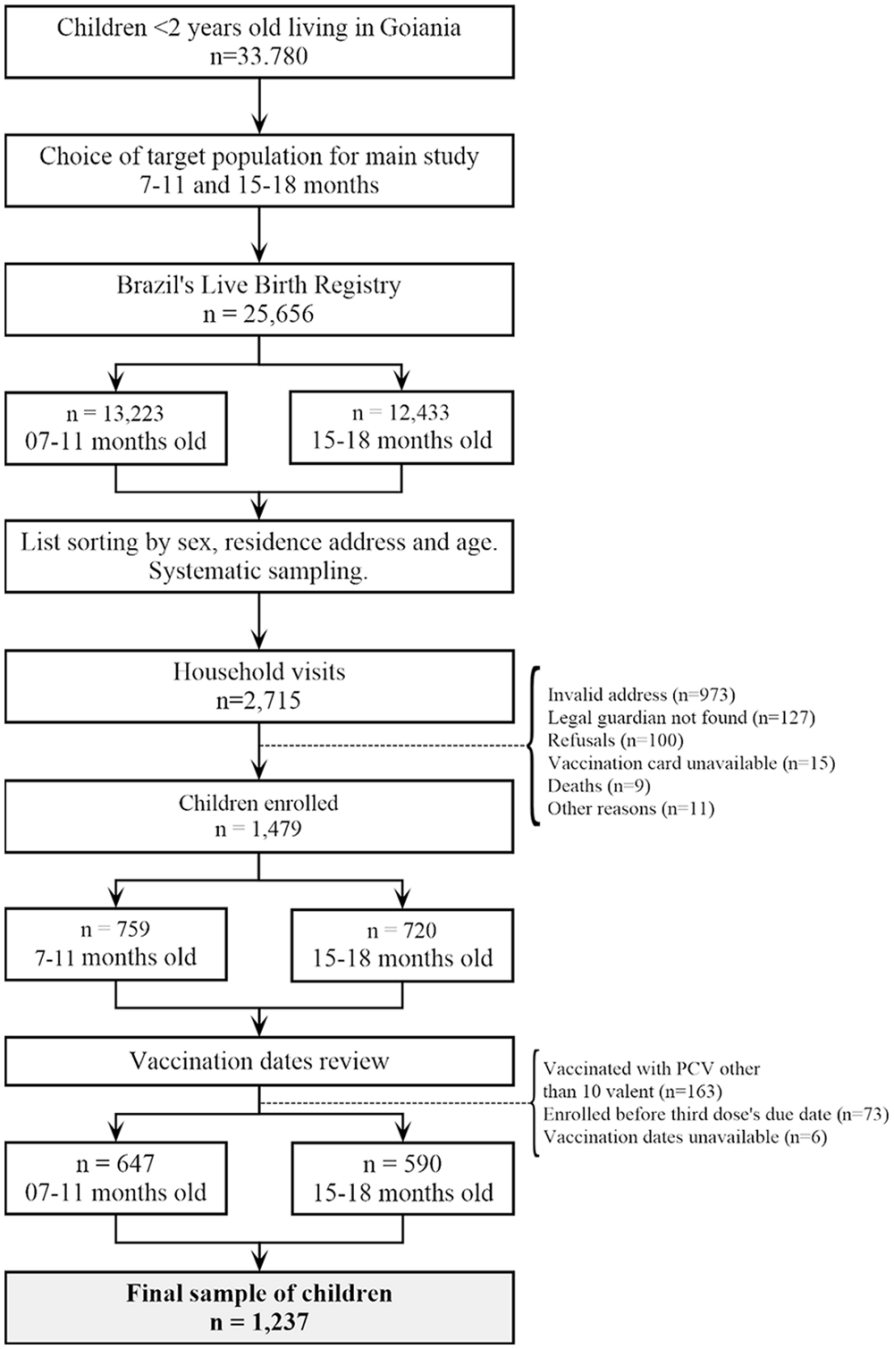
Flowchart of children selection. Goiania, December 2010 to February 2011.

After 2,715 home visits 1,479 children were recruited, of which 1,237 (83.6%) remained in the final sample; the selection process and criteria for exclusion are detailed in Fig 2.

Among the 1,237 participants, 52.4% were male, 64.1% of families did not have private health insurance, and only 5.6% of children attended daycare. As for the mothers, 54.0% went to high school. The following medians were observed: four persons per household, two children in the household, and two people sharing a bedroom with the child. The median annual family income was US$ 8,722.55 (interquartile range US$ 5,472.97–13,682.43).

Only 92 (7.4%) children were not vaccinated at all (95% CI 6.0–8.9), thus being classified as both not covered and non-compliant with PCV10. Of those, 23 had been to a health care service after PCV10 introduction at least once to receive DTP-Hib (See S2 Table. DPT-Hib situation versus PCV10 vaccination status). For children who received one or more doses of PCV10, the median age for the first dose was 4 months for children ≤6 months; 11 months old for children 7–11 months; and 13 months for children 12–15 months old. We found that 837 (73.1%) had received the first dose before 12 months of age. A total of 41 children received more than the recommended number of PCV10 doses, as follows; 33 children received one extra dose (32 from age-group 15–18 months), four received 2 extra doses (all belonging to age-group 15–18 months), and four received three extra doses (all belonging to age-group 15–18 months).

**Fig 3:**
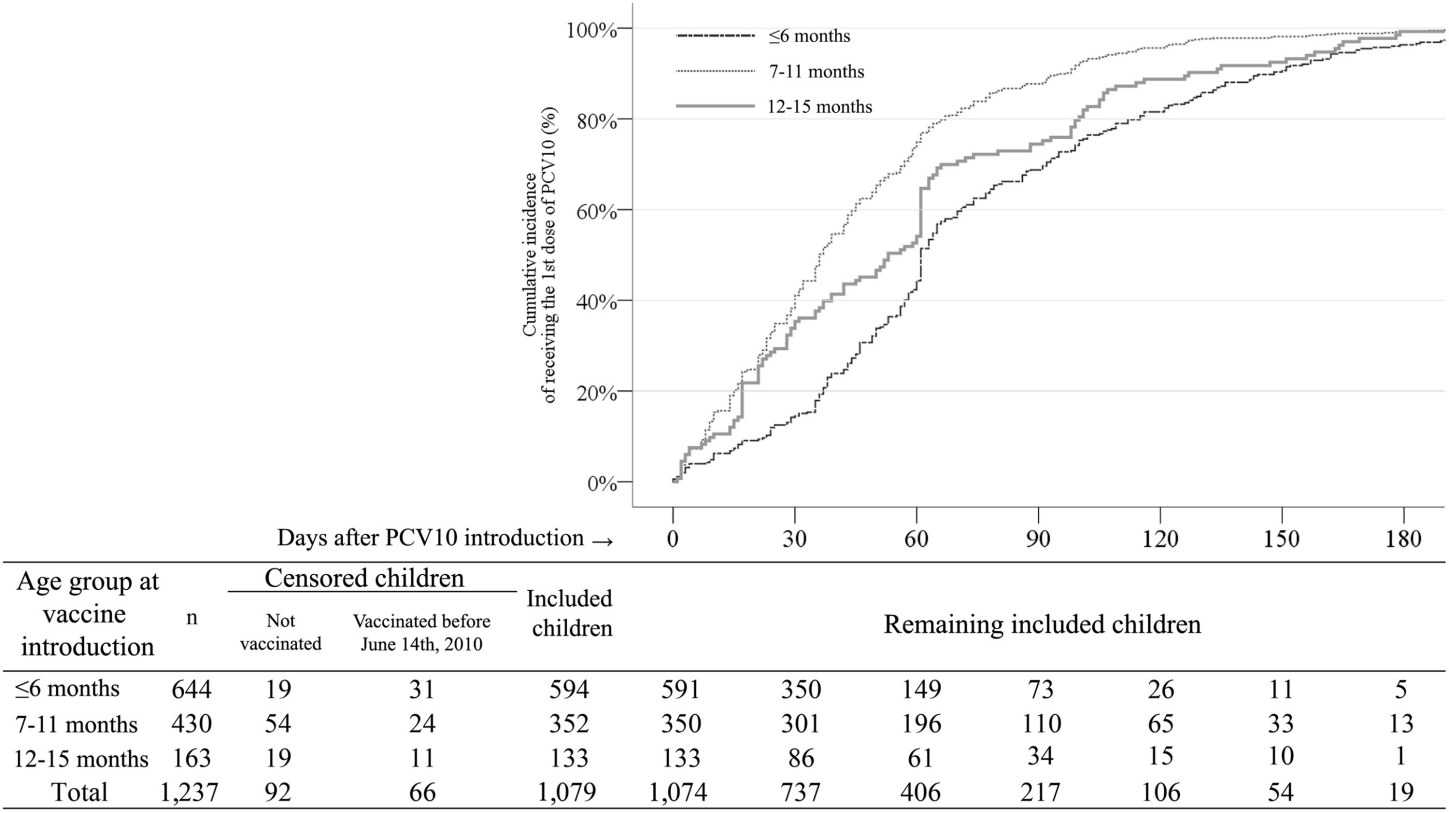
Kaplan-Meier curves of incidence for the first dose of PCV10 after its introduction in the routine immunization program. Goiania, December 2010 to February 2011.

After PCV10 introduction, the median time to receive the first PCV10 dose was 36 days for children ≤6 months (SE 3.4; 95% CI 46.3 to 59.7); 61 days for 7–11 months (SE 1.1; 95% CI 58.9 to 63.1), and 53 days for children 12–15 months of age (SE 3.4; 95% CI 32.8 to 39.2). When comparing the three age groups, the difference among their cumulative incidence curves for the first PCV10 dose was significant (χ2: 105.6; p <0.001) (Fig 3).

Overall vaccination coverage was 53.4% (95% CI 50.8–56), lower than DTP-Hib coverage (93.0%; 95%CI: 91.5–94.3%) (See S1 Table). A significant difference in PCV10 coverage was found among age groups (χ^2^ = 116.9; p<0.001). Children 7–11 months old had the lowest coverage (39.3%), when compared with children ≤6 months old (54.0%) and children 12–15 months old (88.3%), as shown in Table 1. Altogether, 484 (39.2%) children were under vaccinated: 287 received one dose and 197 received two doses. We identified 216 children who received all three doses of DTP-Hib after PCV10 introduction. Of those, 48 failed to complete the recommended PCV10 schedule (see S2 Table); had these opportunities not been missed, coverage would increase 3.9%.

**Table 1:** Vaccination status according to doses and age groups, 6 to 8 months after PCV10 introduction.

Status	Age group at vaccine’s introduction^a^							
	≤6 months old (n = 644)		7–11 months old (n = 430)		≥12–15 months old (n = 163)		Total (n = 1,237)	
	N	%	n	%	n	%	n	%
Received the 1st dose	625	97.0	376	87.4	144	88.3	1145	92.6
Received the 2nd dose	545	84.6	168	39.1	21	12.9	734	59.3
Received the 3rd dose	348	54.0	20	4.7	8	4.9	376	30.4
Received the booster dose	0	0.0	1	0.2	4	2.5	5	0.4
Under vaccinated	277	43.0	207	48.1	0	0.0	484	39.1
Received extra doses	0	0.0	21	4.9	32	19.6	53	4.3
PCV10 coverage (fully vaccinated)	348	54.0	169	39.3	144	88.3	661	53.4
95% CI for PCV10 coverage (%)	(50.3–58.0)		(36.1–45.5)		(83.6–92.8)		(50.8–56.0)	

Goiania, December 2010 to February 2011.

^a^Age was calculated for July 14th, 2010, 30 days after vaccine introduction on routine immunization.

Tolerance for socioeconomic variables varied from 0.752 to 1.00 and no collinearity was observed. All variables were associated with being fully vaccinated but two: gender and daycare attendance. After adjustment, vaccination coverage PR remained significantly higher for the following variables: ≤2 persons sharing a bedroom with the child, and having private health insurance (Table 2).

**Table 2:** Variables associated with PCV10 coverage, 6 to 8 months after its introduction.

Variables	Vaccination coverage*									
	Fully vaccinated		Under or not vaccinated							
	n	%	n	%	PR	95%CI	p	Adj PR	95%CI	p
Gender										
Male	354	54.6	294	45.4	1.05	(0.94–1.16)	0.377	-	-	-
Female	307	52.1	282	47.9						
Mother’s schooling										
High school or above	493	56.7	376	43.3	1.24	(1.09–1.41)	<0.001	1.06	(0.92–1.22)	0.431
Up to elementary school	165	45.8	195	54.2						
Household members										
≤4 people	455	56.7	348	43.3	1.19	(1.06–1.34)	0.002	1.10	(0.94–1.28)	0.232
≥5 people	205	47.5	227	52.5						
Household children										
≤2 children	596	55.5	478	44.5	1.40	(1.15–1.70)	<0.001	1.13	(0.94–1.36)	0.192
≥3 children	64	39.8	97	60.2						
People sharing child’s bedroom										
≤2 people	571	56.4	442	43.6	1.37	(1.15–1.63)	<0.001	1.18	(1.02–1.37)	0.024
≥3 people	81	41.1	116	58.9						
Family’s annual income (US$)										
≥8,722.55	319	58.1	230	41.9	1.21	(1.09–1.35)	0.001	1.11	(0.96–1.27)	0.167
<8,722.55	285	47.9	310	52.1						
Type of health insurance										
Private	266	62.3	161	37.7	1.27	(1.15–1.41)	<0.001	1.22	(1.04–1.43)	0.013
Public	388	48.9	405	51.1						
Daycare attendance										
Yes	42	60.9	27	39.1	1.15	(0.94–1.40)	0.203	-	-	-
No	618	53.0	548	47.0						

Goiania, December 2010 to February 2011.

PR: prevalence ratio; adj PR: adjusted prevalence ratio.

* The sum of individuals is not always 1,237 because there are missing answers, but that represents less than 8% of all participants.

Compliance with the recommended schedules was 16.6% (95%CI: 14.5 to 18.7%); 940 children (76%; 95%CI 73.6–78.4) delayed the first and/or subsequent doses and no child received an invalid dose.

The difference in compliance with recommended schedules among the age groups was statistically significant (χ^2^ 79.3; p<0.001): 18.8% for children ≤6 months old (95%CI: 15.8 to 21.9%), 6.0% for children 7–11 months old (95%CI: 4.0 to 8.5%) and 35.6% for children 12–15 months old (95%CI: 28.5 to 43.3%) (Table 3).

**Table 3:** Compliance with PCV10 recommended schedules by dose and age groups, 6 to 8 months after its introduction.

Status	Age group at vaccine’s introduction^a^							
	≤6 months old (n = 644)		7–11 months old (n = 430)		≥12–15 months old (n = 163)		Total (n = 1,237)	
	n	%	n	%	n	%	n	%
Compliant with the 1st dose^b^	367	57.0	75	17.4	58	35.6	500	40.4
Compliant with the 2nd dose	340	52.8	84	19.5	-	-	424	39.5^c^
Compliant with the 3rd dose	190	29.5	-	-	-	-	190	29.5^d^
Invalid doses	0	-	0	-	0	-	0	-
Delayed one or more dose	504	78.3	350	81.4	86	52.8	940	76.0
Compliant with schedule^e^	121	18.8	26	6.0	58	35.6	205	16.6
95% CI for compliance (%)	(15.8–21.9)		(4.0–8.5)		(28.5–43.3)		(14.5–18.7)	

Goiania, December 2010 to February 2011.

^a^ Age was calculated for July 14th, 2010, 30 days after vaccine introduction on routine immunization.

^b^ For 207 of the participants, the deadline for the 1st dose was the day they reached two months + 30 days of age, because that took place after July 14th, 2010.

^c^ The 2nd dose was recommended for 1,074 of 1,237 children (age groups ≤6 and 7–11 months old), and percentage was calculated accordingly.

^d^ The 3rd dose was recommended for 644 of 1,237 children (age group ≤6 months old), and percentage was calculated accordingly.

^e^ Children who received all valid doses, within the time interval recommended by the National Immunization Program.

Tolerance varied from 0.749 to 1.00 and no collinearity was observed. All variables but three were associated with being compliant with recommended schedules: gender, number of household members and daycare attendance at univariate analysis. The adjusted prevalence ratio remained significantly higher for having private health insurance (Table 4).

**Table 4:** Variables associated with compliance with recommended PCV10 schedules, 6 to 8 months after PCV10 introduction.

Variables	Compliance Status^a^									
	Compliant		Delayed or not vaccinated							
	n	%	n	%	PR	95%CI	p	Adj PR	95%CI	p
Gender										
Male	109	16.8	539	83.2	1.04	(0.77–1.40)	0.805	-	-	-
Female	96	16.3	493	83.7						
Mother’s schooling										
High school or above	159	18.3	710	81.7	1.50	(1.10–2.04)	0.009	1.01	(0.96–1.07)	0.695
Up to elementary school	44	12.2	316	87.8						
Number of household members										
≤4 people	138	17.2	665	82.8	1.11	(0.85–1.45)	0.450	-	-	-
≥5 people	67	15.5	365	84.5						
Number of household children										
≤2 children	191	17.8	883	82.2	2.05	(1.22–3.43)	0.004	1.06	(0.99–1.13)	0.080
≥3 children	14	8.7	147	91.3						
Number of people sharing child’s bedroom										
≤2 people	181	17.9	832	82.1	1.60	(1.06–2.42)	0.021	1.05	(0.99–1.12)	0.095
≥3 people	22	11.2	175	88.8						
Family’s annual income (US$)										
≥8,722.55	104	18.9	445	81.1	1.34	(1.03–1.75)	0.028	1.03	(0.97–1.09)	0.341
<8,722.55	84	14.1	511	85.9						
Type of health insurance										
Private	97	22.7	330	77.3	1.60	(1.06–2.42)	<0.001	1.08	(1.01–1.15)	0.019
Public	106	13.4	687	86.6						
Daycare attendance										
Yes	13	18.8	56	81.2	1.14	(0.69–1.90)	0.607	-	-	-
No	192	16.5	974	83.5						

Goiania, December 2010 to February 2011.

PR: prevalence ratio; adj PR: adjusted prevalence ratio.

^a^ The sum of individuals is not always 1,237 because there are missing answers, but that represents less than 8% of all participants.

## Discussion

This study assessed PCV10 coverage and compliance with recommended schedules in Brazil early at the start of vaccination. The overall PCV10 coverage was 53.4%. This might appear low at first glance, since the Brazilian NIP is recognized for its high rates of vaccination coverage [6]. However, this is a major increase compared to the pneumococcal vaccine coverage of 7.2% observed by a national survey in 2007 [17] when other PCVs were available for free for children with special needs only or to all children who could afford it from private services. Very few studies have assessed PCV vaccination coverage shortly after the start of routine immunization. In the United States, the first pneumococcal conjugated vaccine was PCV7 in 2000. Three quarters after its introduction, a National Immunization Survey on PCV7 observed that coverage with 3 or more doses was 28.3% for children at 7 months of age [23], lower than the 54.1% coverage observed for children aged ≤6 months, who were recommended the same schedule.

Coverage of PCV10 was lower than that of DTP-Hib in our study. However, DTP-Hib has traditionally been part of the Brazilian routine immunization and it is no surprise that its coverage was high in our population. Shortly after their introduction, coverage of pneumococcal vaccines was lower than DTP/DT/DTaP-Hib immunization in other countries. PCV coverage progressively increased for some years before it matched DTP/DT/DTaP-Hib coverage, even though both vaccines were administered concurrently [24–28]. A similar evolution in PCV coverage is expected to take place in Brazil. Nevertheless, the challenge remains, which is, to accelerate this process.

When comparing the uptake of PCV10 and DTP-Hib, we identified a relatively small number of missed opportunities. In Brazil, almost all vaccination rooms are under the responsibility of nurse professionals; the majority of children come to the service without a prescription (since routine immunization does not require that), and it is up this professional to choose the vaccines to be given, based upon current guidelines. Zhu and cols [29] observed that nurses are subject to making mistakes when selecting the appropriate vaccine: they might miss the opportunity, or not properly validate the patient’s vaccination history, or give an extra dose. A clinical decision support system could be useful to decrease human error, especially when recommendations are changed, for instance, by the introduction of a new vaccine.

Also, it is worthy of notice that, even though PCV10 coverage for the complete schedule was comparably low, the percentage of children who received at least one dose of PCV10 was very close to DTP-Hib coverage. Domingues and cols [30] have demonstrated that single catch-up dose in children aged 12–23 months was effective against vaccine-type disease in Brazil. Therefore, even though PCV10 coverage was not as high as traditional vaccines, the fact that the majority of children was indeed vaccinated in such a short time is in any case, valuable.

This study clearly reveals significant disparities in compliance, an important gap in Brazil’s NIP performance. This is consistent with other studies in low, median and high income countries, which have demonstrated that assessing routine immunization through coverage estimates alone hides shortfalls in compliance [12, 31, 32]. In our study, most non-compliant children had delayed one or more doses, which might explain why almost 40% of children failed to complete the recommended schedule. Any delay on vaccination may have a great impact, since it unnecessarily prolongs exposure to pneumococcal diseases, which are a leading cause of morbidity and mortality in infants [1].

Brazil’s NIP opted for different vaccination schedules at the introduction of PCV10 routine immunization, one for each of the first three semesters of life, beginning with three primary doses and dropping one primary dose from semester to semester (catch-up schedules). Higher costs and logistics that were more complex than using a single schedule presumably ensued from this decision. One important finding of the present study was that the median time to start vaccination, vaccination coverage, and compliance with recommended schedules were different among the three age groups / schedules. Children 7-11months old (who received two primary doses) had the highest median time for the first dose, the lowest vaccination coverage and the lowest compliance, when compared to children aged 12–15 months (single dose) and ≤6 months (three doses).

The vaccination calendar adopted in Brazil [7] might explain our results. The considerable number of vaccinations and of routine medical appointments at the first semester of life and at the first birthday would get children to health services more often, thus facilitating the contact with a new vaccine such as PCV10, which could explain the higher coverage and compliance at the age groups of ≤6 months and 12–15 months old children. At the second semester of life, the number of appointments is reduced to one single growth follow-up visit and one vaccine (yellow fever) at 9 months of age, which the child’s family might not always seek. The low PCV10 coverage and compliance for children at the second semester of life could reflect it. This is cause for concern because, in Brazil, the incidence of pneumococcal disease is higher precisely in the second semester of life [33].

Because both compliance and coverage were so distinguished among the three age groups, the results of this investigation suggest that, during the introduction period, if a vaccine has different schedules for different age groups, each age group must be evaluated independently in order to properly estimate the gaps in vaccination coverage and compliance.

The NIP mainly focuses on reaching high vaccination coverage among the target population, and compliance of vaccination is not routinely evaluated. In this study, delaying one or more doses of PCV10 was frequently observed at all age groups and for all doses. This result suggests that the NIP goal should be shifted to achieving high levels of protection as early as possible; high coverage rates will follow. In addition, it would be highly desirable to include compliance indicators in regular NIP performance evaluations.

Surprisingly, having private health insurance was associated with having completed the number of recommended doses as well as having received all doses in the recommended time interval. These observations may seem contradictory since PCV10 was available for free exclusively from public health providers. One possible explanation is that the availability of pneumococcal vaccines on private providers before its introduction on routine immunization created a pent-up demand for them among privately insured families [6]. Once the National Immunization Program included PCV10 on routine immunization, holders of private health insurance could vaccinate their children at public (free of charge) providers, instead of paying for them in private providers. Vaccination costs is thus transferred to the Brazilian Unified Health System (SUS), which already funds immunization almost singlehandedly [34]. Also, they seek health care more regularly than those without insurance and they are likely to attend clinics and outpatient services (where vaccination takes place) more often than people who do not have insurance, who tend to seek care only when ill, mostly at emergency rooms [27]. Further research would be necessary to confirm if differences in health care access or behavior are behind the higher PCV10 prevalence of coverage and compliance observed for private insurance holders.

Efforts to reach under-vaccinated and/or non-compliant children should be pursued with a multifaceted approach [9, 29, 35, 36]. Because families without private health insurance tend to look for care only when the child is ill, any visit to health services, including to the emergency room, should be used to update the child’s vaccination status, if the reason that brought the child to the service does not contraindicate vaccination. Therefore, delays could be promptly identified and resolved [37–40].

Limitations of this study should be mentioned. Some variables that are known to be related to inadequate vaccination could not be analyzed because they were not collected for the major study [9, 35, 41–43]. Due to the short time elapsed between the introduction of PCV10 and data collection, it was not possible to compare coverage and compliance of schedules of 2 and 3 primary doses without the booster dose to schedules of 2 and 3 primary doses with the booster dose. Another issue is that in actual fact, we cannot fully extrapolate the findings to children outside our recruitment age ranges. As we saw that coverage increases with increasing age, children below our range are likely to have poorer coverage and those above the range are likely to have greater coverage. Finally, the choice of the target population for enrollment for the major study did not take into account all children at the age group of 12–15 months old. However, because the size of the final sample was much larger than it would be necessary for the purpose of the present study, it is unlikely that this limitation compromised the estimation of coverage or compliance in this age group.

This study was conducted shortly after PCV10 introduction, which took place five years ago. It is feasible to say that the transition period is currently over, there is only one cohort of unvaccinated children and the primary doses of PCV10 are routinely administrated for children at 2, 4 and 6 months of age; other schedules are likely to be used only when the child missed vaccination. In this context, new studies should be conducted to assess PCV10 coverage and compliance in order to see whether coverage has reached Brazil’s NIP 95% goal and how children are complying with this schedule, now that PCV10 is included in the routine immunization just like other vaccines in the childhood vaccination calendar.

In conclusion, a significant percentage of PCV10 coverage was achieved in a relatively short time, but it was not followed by compliance with recommended schedules. There is room for improvement. Public initiatives in Brazil should target compliance of PCV10 because of the burden of pneumococcal diseases on childhood morbidity and mortality, especially at the second semester of life, where the risk of such diseases is highest, and both coverage and compliance are lowest.

## Supporting Information

S1 Table DTP-Hib vaccination status according to age group at PCV10 introduction. Goiania, December 2010 to February 2011.
(DOCX)

S2 Table DTP-Hib situation versus PCV10 vaccination status. Goiania, December 2010 to February 2011.
(DOCX)
