# Supplementary material for: Vaccination Coverage and Compliance with Three Recommended Schedules of 10-Valent Pneumococcal Conjugate Vaccine during the First Year of Its Introduction in Brazil: A Cross-Sectional Study
Source: PLoS One. 2015 Jun 10;10(6):e0128656. doi: 10.1371/journal.pone.0128656 (PMC4489587; doi:10.1371/journal.pone.0128656)
Supplement: S1 Table — Goiania, December 2010 to February 2011. (DOCX) [file pone.0128656.s001.docx]

**S1 Table. DTP-Hib vaccination status according to age group at PCV10 introduction.** Goiania, December 2010 to February 2011.

| Status for  DTP-Hib vaccination | Age group at PCV10 introduction^a^ | | | | | | Total  (n=1,237) | |
| --- | --- | --- | --- | --- | --- | --- | --- | --- |
|  | ≤6 months old  (n=644) | | 7-11 months old  (n=430) | | 12-15 months old  (n=163) | |  |  |
|  | n | % | n | % | n | % | n | % |
| Received the 1st dose | 634 | 98.4 | 418 | 97.2 | 161 | 98.8 | 1,213 | 98.1 |
| Received the 2nd dose | 620 | 96.3 | 413 | 96.0 | 161 | 98.8 | 1,194 | 96.5 |
| Received the 3rd dose (completed the schedule) | 577 | 89.6 | 413 | 96.0 | 160 | 98.2 | 1,150 | 93.0 |
| Under vaccination | 58 | 9.0 | 7 | 1.6 | 2 | 1.2 | 67 | 5.4 |
| Unvaccinated | 9 | 1.4 | 10 | 2.3 | 1 | 0.6 | 20 | 1.6 |

a. Age was calculated for July 14th, 2010, 30 days after PCV10 introduction on routine immunization.
