# Supplementary material for: Vaccination Coverage and Compliance with Three Recommended Schedules of 10-Valent Pneumococcal Conjugate Vaccine during the First Year of Its Introduction in Brazil: A Cross-Sectional Study
Source: PLoS One. 2015 Jun 10;10(6):e0128656. doi: 10.1371/journal.pone.0128656 (PMC4489587; doi:10.1371/journal.pone.0128656)
Supplement: S2 Table — Goiania, December 2010 to February 2011. (DOCX) [file pone.0128656.s002.docx]

**S2 Table. DTP-Hib situation versus PCV10 vaccination status.** Goiania, December 2010 to February 2011.

| DTP-Hib vaccination status  in relation to the introduction of PCV10 | PCV10 vaccination Status | | |
| --- | --- | --- | --- |
|  | Full vaccination  (n=661) | Under vaccination  (n=484) | Unvaccinated  (n=92) |
| Received at least one dose of DTP-Hib after PCV10 introduction (n=672) | 361 | 288 | 23 |
| Received all three doses of DTP-Hib after PCV10 introduction (n=216) | 168 | 48 | 0 |
